# Synthesis, Processing and Applications of Conjugated Oligomers and Polymers 2.0

**DOI:** 10.3390/ijms241411623

**Published:** 2023-07-19

**Authors:** Dario Pasini, Szczepan Zapotoczny

**Affiliations:** 1 Department of Chemistry and INSTM Research Unit, University of Pavia, Via Taramelli 12, 27100 Pavia, Italy; 2 Faculty of Chemistry, Jagiellonian University, Gronostajowa 2, 30-387 Kraków, Poland

In the past few decades, conjugated organic oligomers and polymers have been shown to have amazing properties, such as conductivity, which were traditionally considered counterintuitive for macromolecules consistently used as plastics and fibers (and thus, insulators) until the late 1970s. Their semiconducting properties have garnered an enormous amount of interest, and indeed, they have ushered in a new generation of materials, often pairing the outstanding performances of inorganic semiconductors with advantages in terms of their relative cost, their straightforward processing methodology and their fields of applications. They are now widely used in sensing, energy harvesting and conversion and electronics due to their amazing optical, electronic, chemical and mechanical properties. Because of their semiconducting properties, they have been associated with three main technologies, often referred to as the “big three” of plastic electronics: organic field effect transistors (OFETs), organic solar cells (OSCs) and organic light-emitting devices (OLEDs).

Solution-processable, π-conjugated polymers, which intrinsically possess suitable mechanical properties linked to their macromolecular structure, allow the use of competitive, cost-effective fabrication techniques, such as spin coating or continuous roll-to-roll, for large-area applications. The vapor phase deposition of an organic material thin film was initially reported in the literature as the technique of choice for small molecules; alongside π-conjugated polymers, the development of π-conjugated small molecules with similar and suitable filmability and processability characteristics has been an area of significant interest in recent years. The discrete and well-defined structures of such small-molecule materials, which can be prepared in high purity with excellent synthetic reproducibility, avoids the batch-to-batch differences which unavoidably occur in polymer synthesis.

Although conjugated materials are booming and affecting many important fields, they still face some key challenges, mainly in terms of their time-consuming synthesis and low yield, their limited applications because of instability and their poor solubility. Therefore, the long-term development of conjugated organic materials urgently requires multidisciplinary researchers to work together; in operando spectroscopic techniques are a good match for organic, polymer chemistry, and engineering; therefore, they are always the basis of breakthroughs in the field. Effective and scalable synthesis will be an increasingly important driving force for the long-lasting vitality of conjugated materials [1,2].

The Special Issue entitled “Synthesis, Processing and Applications of Conjugated Oligomers and Polymers 2.0”, published in the *International Journal of Molecular Sciences,* includes a total of six contributions, all of them being original articles, which provide a broad panoramic view of recent scientific activities in the synthesis and characterization, including the application, of π-conjugated oligomers and polymers.

Lin, Liu et al. [3] reported the synthesis several novel thiophene-isoindigo–thiophene D–A–D-type oligomers, obtained through Stille coupling, and their electropolymerization to yield macromolecules with interesting electrochemical and electrochromic performances. Isoindigo and thiophene moieties are amongst the prototypical systems, as π-electron acceptors and π-electron donors, respectively, developed in the field of organic π-conjugated semiconductors, yet this paper presents a clear advancement over the state of the art, as the combination of the electropolymerization strategy with D–A–D-type polymers is not common. The contribution thoroughly addresses the structure–property relationships of the precursors and corresponding polymers through a variety of measurements such as surface morphology, band gaps, electrochemical properties and electrochromic behaviors. The materials combine the merits of isoindigo and polythiophene, including the excellent stability of isoindigo-based polymers and the extraordinary electrochromic stability of polythiophene. The isoindigo–thiophene D–A–D-type polymers possess favorable electrochromic performances, including high optical contrast (53%, 1000 nm), a fast switching time (0.8 s), and high coloration efficiency.

Chang et al. [4] reported that the incorporation of graphitic carbon nitride (g-C_3_N_4_) into a conjugated polymer matrix can improve the sensing performance of OFET gas sensors, which have recently attracted considerable attention for use in environmental monitoring applications. The existing devices are limited by their poor sensing performance for gas analytes, probably due to the low charge transport and the limited charge–analyte interaction of the conjugated polymers. Here, g-C_3_N_4_ was tested systematically in combination with poly(3-hexylthiophene) (P3HT) by changing its concentration in the P3HT/g-C_3_N_4_ composite films. The obtained films were applied to detect NO gas at room temperature. Charge carrier mobilities of ~1.1 × 10^−1^ cm^2^ V^−1^ S^−1^, a five times enhancement over pristine P3HT films, could be obtained, and the gas sensing characteristics were also considerably improved; for example, when exposed to 10 ppm NO gas, responsivity increased to 40.6 from 18.1%, with a response time of 129 s from 142 s.

Angel et al. [5] reported on novel conjugated terpolymers, with benzodithiophene and benzotriazole units and bearing a fluorescein derivative as a side group, which were covalently bounded to the backbone through a saturated, flexible n-hexyl chain to induce the intramolecular Förster Resonance Energy Transfer (FRET) process and possibly improve the photovoltaic performance of the polymeric material in organic solar cell (OSC) applications. Photophysical measurements have demonstrated the occurrence of the FRET phenomenon between the lateral group and the terpolymer. The terpolymer exhibited an absorption band centered at 501 nm, resulting in an optical bandgap of 2.02 eV, and HOMO and LUMO energy levels of −5.30 eV and −3.28 eV, respectively, which are not optimal for OSC applications. However, preliminary studies showed that FRET-based devices performed better when compared to those based on an analogous polymer without the fluorescein derivative. These results are therefore encouraging for making improvements in the performance of polymers used in OSCs through the FRET approach.

Lin et al. [6] reported on the synthesis of novel oligomers capable of functioning as nonfullerene acceptors for OSCs. Several systems with the organic moiety truxene at the center, endcapped with 3-ethylrhodanine, as electron acceptors for OSCs with high absorption coefficients, were prepared and tested. The authors performed a large series of syntheses and experiments to optimize the novel compound series’ performances in cells, including approaches towards energy levels, morphology, alkyl chain (hexyl/decyl) and branched-arm engineering (three/six branched arms). Although the power conversion efficiencies (PCEs) were rather low, the value was obtained without additional additives and post-processing, therefore broadening the potential applications of star-shaped truxene building blocks in the fields of organic electronics.

A stepwise methodology for the synthesis of surface-grafted conjugated polymer brushes was reported by Zapotoczny et al. [7]. Alternating donor–acceptor chains were grown using selected cross-coupling reactions and properly derivatized bithiophene (electron-donating) and benzothiadiazole (electron-accepting) groups. Applying Sonogashira coupling, brushes with thicknesses of ca. 13 nm were obtained. Their semiconductive properties and reduced bandgap (E_g_ ≈ 2.34 eV) make them promising ordered layers for photovoltaic and optoelectronic application, and the method may be easily adapted to obtain polymer brushes of other defined sequences of mers.

Zapotoczny et al. [8] also reported on polymer brushes containing poly(propylenedioxythiophene) conjugated chains in a ladder-like architecture with improved (photo)stability as compared to polythiophene-based brushes. The photophysical properties of such novel brushes were thoroughly characterized, which provided information about the effective conjugation lengths of the segments within the grafted chains. The results indicated that such conjugated polymers, although difficult to process in solution, may be formed in situ on a surface, providing a platform for, e.g., electronic or sensing applications.

This Special Issue aims to be an up-to-date and comprehensive issue focused on conjugated materials and aims to pave the way for future practical applications in this wide and dynamic field. We believe it will be an interesting and worthwhile read for all scientists belonging to the chemistry, material science and applied physics community.
